# The Factors Concerned in Dental Deterioration

**Published:** 1892-07

**Authors:** L. C. F. Hugo

**Affiliations:** Washington, D.C.


### THE FACTORS CONCERNED IN DENTAL DETERIORATION.[1]

BY L. C. F. HUGO, D.D.S., WASHINGTON, D. C.

Our patients often ask us why it is that human teeth are so bad and so much worse than those of older generations.

With a view to being prepared with an answer, in accordance with our present light, I have consulted on this subject the best thought of our profession. In this paper I present to you a *résumé* of that inquiry, together with some comments and elaboration of my own.

Though limited to two or three generations, the layman's observation of the progressive decadence of civilized man's dental organs is in harmony with the dentist's extended knowledge covering thousands of years.

According to Dr. Darby, of Philadelphia, the teeth of the ancient monks in the charnel-house on Mount Sinai show that the farther back they date the more nearly free from decay they are. He examined a large number of Egyptian mummies, finding ample jaw and but little defective dental structure. The remains found in the *huacas* or tombs of Peru show the teeth in almost perfect condition, as is likewise the case with those in the crypt at Hythe, England. The Druids of Anglesea, as attested by their teeth, were seldom subject to dental troubles.

In disagreement with the examples of ancient teeth just given are the Etrurian remains, which show extensive dental lesions. To my mind the condition of these teeth does not antagonize, but is rather a confirmation of, the opinion that among civilized nations

---

[1] Read on the twenty-fifth anniversary, November 23, 1891, of the Washington City Dental Society.

there is progressive dental deterioration. Let us see. Hundreds of years before the founding of Rome, Etruria was a *civilized* country. Commerce was the principal pursuit of its inhabitants. A luxurious life was the goal of their existence. Furthermore, the teeth, having been found in tumuli, are in all probability the remains of a class of persons in whom ease, luxury, vice, capricious appetites had wrought the same results as are now observed under similar circumstances among us. The Etrurian examples are only apparently exceptional.

Now as to modern teeth. Dr. Beers, of Montreal, says, "It is probably a perfectly safe estimate to make that in American and Canadian cities of fifty thousand people not a hundred native-born can be found, between the ages of four and fourteen, who have wholly escaped caries and premature loss of some of the teeth. If the ravages were to proceed in the same ratio, men and women would be edentulous by the time they were forty, were it not for the preservative skill of the dental operator." Dr. Ottofy, of Chicago, examined the teeth of children in public schools. Of six hundred and twenty-three pupils between the ages of five and fifteen, only forty-eight had perfectly sound teeth,—that is, only seven and one-half per cent.

Dr. B. W. Richardson, an English observer, "found that, of over four thousand persons of both sexes and of all ages, over eighty, per cent. were affected more or less severely with dental caries, while it was rare to meet with a person in whom both sets of teeth were altogether free from disease."

Though this is the extreme of widespread dental defectiveness, it may serve as an illustration of what is found among modern civilized nations, and among semi-civilized or barbarian people that come in prolonged contact with civilization.

Not only has there become observable in the teeth a gradual structural change, but an anatomical one as well. It has been noted that frequently the upper wisdom-teeth of civilized nations have only two roots,—and these often coalesced,—whereas the *dens sapientiæ*, as seen in the most ancient jaws exhumed in Europe, shows uniformly three distinct roots. Nature does not stop here, but, in accordance with "the law of economy of growth," she is stamping condemnation upon the tooth under consideration, by permitting it to be of readily decayable structure, or by suppressing it altogether. The upper lateral, too, is occasionally missing. These two classes of teeth are the least useful in the set, and in a crowded jaw they may well be dispensed with to give their better neighbors more room. The question presents itself—*Quousque tandem?*—will this process of elimination end here?

Let us now examine the factors concerned in bringing about man's dental impairment.

For convenience of consideration these factors may be grouped as *remote*,—those affecting the teeth through the general system, whether directly or indirectly,—and *immediate*,—those directly affecting the external local condition of the teeth.

The *remote factors* are,—

1. Civilization in some of its *effects or concomitants.*
2. Change of diet involving a lack of functional exercise of the teeth.
3. Impairment of the general health.
4. Prenatal influences.
5. Crossing.

CIVILIZATION IN SOME OF ITS EFFECTS OR CONCOMITANTS.

It is an indisputable fact that certain features of civilized life tend to the decadence of physical vigor. The principal element in this condition, however, is the check offered by civilization to the weeding-out process of "the survival of the fittest." The healing art and improved sanitary observance have kept and are keeping alive persons that in uncivilized life would have died before reaching maturity. It is the offspring of this class of persons that furnishes so large a percentage of the sum total of physical degeneracy. "For all France the mortality in 1771 was 34" (in 1000); "by the year 1801 it had dropped to 28; in 1883 to 22." This shows an immense gain in the preservation of life,—the weak deriving the larger share of the gain.

Though civilization has done this much to preserve and propagate the physically degenerate, and though it has decidedly enervating influences, we must not—and this mistake is often made—*blame civilization per se.* It is not necessarily incompatible with sound physical development. Some of the most highly civilized people, such, for example, as members of the upper class of European society living in the country, have a strong, sound physique, with good teeth, much better usually than their countrymen of a lower social scale. But these persons are exceptional, because they lead conservative lives; because they live near nature; because they follow Cleobulus's advice, "Avoid excess;" because they take advantage of the *beneficial* features of civilization, and by intelligent discrimination escape the deleterious.

Comparing the nerves, muscles, and bones of the present civilized with those of uncivilized man,—ancient or modern,—we cannot help thinking that, in some degree, the ameliorations of civilized life impose physical degeneracy. By the word ameliorations, as here employed, is meant in a broad sense the supplantation of brawn by brain, bodily exertion by labor-saving machinery, exposure by protection.

Primitive man, with his steady nerves, tough muscles, and strong bones, was by the stimulus of his environment and by the weeding out of the weak, prepared to hold his own under the trying conditions of the struggle for existence. This resulted in the production and preservation of physical soundness.

Man, however, is said to be naturally lazy. His brain from the awakening impulse studied the "royal road" to ease,—that is, he began ameliorating his condition. In course of time, as his intellect developed, his experience increased, his observations expanded, he began to subjugate the animal world and the crude forces of nature, to serve as more convenient and improved means to his ends. In other words, the cunning of his brain devised means to lighten the burden of his muscle. It sought to secure him safety from the wild beasts and from the inclemencies of weather; it sought to widen the range of his food articles, to save an expenditure of effort in making these articles more easily obtainable and less difficult to chew. In short, he began growing away from the very conditions that had developed him into physical perfection, to become that artificial product,—that paradoxical miscellany of virtue and vice, of strength and weakness, *modern civilized man.*

In a great measure he has made himself; hence he lives without due regard to many of the requirements of his animal economy. Quite true, tolerant nature will adapt herself to circumstances. But when man outruns his rate of adaptation, he must suffer impairment of health.

Dental defectiveness was the exception not only in ancient man, where he lived, as Aurelius advises, "according to nature," but it is the exception even now in uncivilized man, where he still lives in barbarian purity. An examination of his mouth, or of the skull of his immediate ancestor, will give abundant proof of what has just been stated. The Earthmen, living in the Kalihan Desert, South Africa, are, it is said, "complete savages, and yet have ideally perfect teeth." John R. Mummery has examined about three thousand skulls of modern barbarians, finding but little decay and few irregularities. Dr. Winder found in one thousand skulls, principally of

Indians, only about fifty defective teeth. The few Eskimo and even the Sandwich Islander remains made a good showing.

Observation goes to prove that the more nearly pure his type and the nearer to original conditions man lives the better his teeth are. But, let him come in contact with certain ways of civilization, and he will in a few generations bear the indubitable physical record of it. In speaking of the Indians in their habitat, an ethnological observer says, "The vices of civilization have not tainted their blood, nor its luxuries sapped their strength." But removed from their natural home and surroundings, they lose their bodily vigor. The Indian boys put to school at Chemawa are soon transformed into the ordinary cage of human ills, among which "the hell o' a' disease" is not the least annoying. But a comparatively short time ago the teeth of the Sandwich Islander were good. Now, having tasted some of the fruits of civilized life, having contracted new habits and diseases, his teeth are deteriorating. Dr. H. Flagg says of the Matto-Grosso Indian, that in his home, where he is at liberty to secure what he likes, he dies with his teeth in. "Take now the same Indian and subject him to what we call civilization. He may be badly nourished, because he is overworked and underfed, or he may be badly nourished because he is overfed and underworked;" at any rate his teeth suffer. The Southern negro, prior to a quarter of a century ago, when he lived on simple, coarse food, worked regularly, and took proper rest, possessed good teeth; but now, as he gets access to a greater variety of food-articles, prepared so as to require little or no hard chewing, works less, and is more irregular in his habits, in short, enjoys some phases, not always of the best, of civilized life, is beginning to show, by a progressive though slow deterioration of his teeth, the effect of his ameliorated condition. Poor "Lo!" as he becomes civilized, eats pie, and drinks fire-water, evinces in the poorer structure of his dental organs the unmistakable signs of a changed life. In all this we can see a distinct relation between the physical condition of man and the degree of his removal from natural environment. I am, of course, speaking in terms of grand averages.

### CHANGE OF DIET INVOLVING A LACK OF FUNCTIONAL EXERCISE OF THE TEETH.

The teeth grew in man's jaw to meet certain requirements of his diet; and they performed their office just as the teeth of the lower animals of to-day, living in a natural condition, still do their whole duty.

Just here the question may be asked, Why is it that dental decay is by no means infrequent among our four-footed friends, and yet they live on practically the same food as their habitat would yield them. It may be worth while to examine this point. Thus far dental lesions have been observed in such *domesticated* animals as horses and cows. In these occasional cases, may not the animals have been treated in violation of the laws governing their natural food-seeking and food-chewing? May they not have been kept in small, ill-ventilated places? May they not have been neglected as to sufficient feeding and watering? May they not have been obliged to subsist on food that in their habitat they would refuse to eat? Very likely, if we investigate their history, we would find that man's interference will account for these lesions in the lower animals as well as for those in his own case. Where dental decay in the lower animals is not due to man's agency, we may look upon it as a *lusus naturæ.*

Civilized man's extended range of food-articles and his improved methods in making them easily masticable result in a partial disuse of the chewing-apparatus.

While a radical change of diet may only affect the condition of the teeth, it is sometimes followed by surprising anatomical alterations. For example, we are told John Hunter fed a gull on grain. The change from a fish diet resulted in the development of a gizzard, which up till then had been in a rudimentary state. This seems to show that the gull of to-day lives on different food from that of his ancestors. Professor Peirce says of a certain species of ant-eater that in the anterior part of this animal's jaw there are no teeth, except occasionally an aborted lateral. Yet the *germs* of the front teeth are always present in the jaw. He infers that, in the distant past, the ancestors of the ant-eater must have lived on food requiring front teeth; and that a change of diet resulted in this suppression.

It is well known what can be accomplished by specially-directed training. How endurance may be increased, how muscles may be strengthened, how lung-capacity may be enlarged, how even the general frame may be further developed. Even sight, hearing, touch are susceptible of improvement.

If, then, by proper exercise capacity can be raised above the individual normal, may it not by lack of due exercise be brought below that normal, and in time to positive degeneration?

We have all experienced that when, for some reason, chewing must for a while be done on one side of the mouth, the other will feel weak and lame. Now, if exercise be curtailed on both sides, we can readily see how a slight weakening may take place. Multiply this by generations, and an appreciable result will be had. This may serve as an illustration of deteriorated function.

I have repeatedly noticed that the teeth of one jaw opposite to spaces left by extraction in the other were badly decayed, while the teeth in the immediate neighborhood which had opponents were perfectly good. This illustrates deterioration of organ from disuse.

To make generalized statement of the above we may say,—

If exercise of function is divorced from organ, the tendency will be towards deterioration of function, and reflexively deterioration of organ. Hence it may be laid down as a law that it is the degree of functional exercise that determines the state of health, working capacity, and structural condition of the organ.

It will be found that where a people is of pure type, does not intermarry too closely, lives on plain food, which to be comminuted requires a positive effort on the part of the oral mill, the teeth will be of good quality. The Scotchman exercises his chewing-apparatus on coarse, hard food; in some parts of Germany the well-known pumpernickel (black bread) thoroughly tests the efficiency of the native grinders; the South Russian, between meals, chews the hard seed of the Turkish sunflower; the North Siberian eats a coarse black bread, and after meals chews spruce-rosin; the Egyptian and the Arab live on coarse barley bread with almost no meat; our Indian has a meat diet with but little bread, which, however, is coarse; the Terra del Fuegian has large, strong teeth which perform their whole duty on very hard food. All these peoples, where they lead a simple life, comparatively free from strong drink and hereditary encumbrance, have but little, if any, use for the dentist. Those in civilization have, of course, greater need of his services. Where, for example, rice is the "staff," supplemented by other soft food, as among Oriental nations, socket-catarrh and decay of the teeth are more frequent.

Of all peoples, we Americans are *said* to have the greatest need of the ministrations of dentistry. With us are to be found almost all the elements concerned in bringing about dental deterioration. By descent we are a great mixture. Our life is a constant nerve-strain. As James Stirling says, "The deepest-rooted cause of American disease is that overworking of the brain and over-excitement of the nervous system, which are the necessary consequences of their intense activity." Our city population soon reaches the condition of Rufus Choate, who, being warned that he would by his overwork soon break down his constitution, replied, "Good heavens, my dear fellow! my *constitution* was all gone years ago, and I'm living on the by-laws." Our food is multifarious and of soft preparation. Even if it were simple and hard, we would be too much pressed for time to chew it. Hence our teeth have a "plentiful lack" of functional exercise. It is no argument to hold up to us the Eskimo, as has been done, and say, Here is a man that lives on blubber, which does *not* tax the dental organs, and yet he has no dental bills to pay. In the first place, this interesting individual's diet extends beyond blubber. He lives on reindeer, geese, seal, walrus, etc. His teeth crack many a small bone. Furthermore, the Eskimo's forefathers from the "time whereof the memory of man runneth not to the contrary," lived on the same food and used their teeth in the same way as their successors now do. And so long as the latter continues to live in barbarian purity, however filthy that may be, so long will they dispense with the services of dentistry.

With us the case is different. Our forefathers lived nearer to nature than we do. One of the conditions that preserved their chewing-apparatus was vigorous exercise on food necessitating it. This condition has become ameliorated.

For generations it has been the profitable study of the miller to produce a finely-ground flour; for generations it has been the remunerative object of the baker to make of this flour a soft, light bread; for generations it has been the ambitious desire of the cook to produce soft preparations of meat and vegetables. This being the case indifferent teeth are sufficient for present requirements.

Do not misunderstand me as to the miller. He is usually misrepresented. He does not materially rob the flour of its nutriment. Investigation proves that in removing the cortical layers of the grain only a slightly nutritious element is lost. The central four-fifths of the grain contains almost all the desirable elements, namely, gluten, starch, etc. The harm done is this, that, in the refining process, our masticating-apparatus is cheated of its work; and, incidentally, that a good stimulus to peristalsis is lost.

Stephen Girard is quoted as saying that teeth are like boys,—when they had nothing to do they fell into bad habits. To keep his dental boys out of mischief he amused them with "hard tack." I have no doubt that our forefathers were fond of chewing the articular ends of small bones. To this day we occasionally meet persons indulging themselves in this direction; and many more would do so if they were not afraid of breaking their teeth. We cannot deny that the exercise would be beneficial.

A dentist, to illustrate the value of using the grinders on hard food, relates the following: "In a family of five children, whose parents . . . had good teeth, the three eldest, whose diet required something more than the average of mastication, have good and dense teeth. The mother, having great faith in bread and milk, insisted on getting a cow, and for twelve years from their infancy the two youngest children lived mainly on bread broken into milk. The result was in the main good health for the two boys, but lamentably inferior and sensitive teeth." The experiment was ended by putting the boys on hard fare, when a great improvement was noted.

Other things equal, tobacco-chewers have better teeth than non-chewers. Why? Not on account of any known virtue in the weed, but because of the exercise involved. Chewing gum, if we stop to reflect, is not so reprehensible a practice. In fact, the young lady may justify her indulgence by claiming that the muscular effort expended is in the line of economic conservation.

### IMPAIRMENT OF THE GENERAL HEALTH.

That there is an interdependent relation between the whole animal body and its parts is an easily-observed fact. Every day the dentist has cases in which he can see how the general health affects the teeth. One way in which they plainly show their sympathy with the lowering of the systemic tone is in the lessened power of resistance they offer to the changed, vitiated condition of the fluids in the mouth and stomach. Catarrhal throat and nervous dyspepsia, for example, make sad havoc in many mouths.

The teeth, being of a structure that has not the power of restoration possessed by the soft tissues, will often bear permanently a record of profound systemic disturbance. Eruptive diseases, such as scarlet fever, small-pox, even measles and enteric affections, will sometimes leave their indelible inscriptions.

In the adult, nerve-wear, brain-strain, will at times so reduce the resistive power of the teeth that the dentist's restorative efforts become despairingly inefficient. Dr. Fothergill says in this connection, "The dental caries so prevalent, indeed universal at the present day, is but a part of the general widespread failure of the digestive organs. . . . Whence comes the profound modification of the organic processes, the commissariat of the active or animal part of the body? It is the effect of modern life upon the nervous system." Dr. J. L. Williams has observed degeneration of dental tissues as "a result of long-continued, intense mental activity."

Gout and rheumatism, with their particular effect upon the alveolo-dental membrane, tubercular, syphilitic, scrofulous taints, morphine habit, in short any affection or practice reducing neural energy which is followed by a disturbance of the nutritive repair, will have its due deleterious effect upon the dental organs. When we consider that in addition to its special troubles, our chewing-apparatus is under the implacable tyranny of hereditary diatheses, it is burdened indeed.

Under the present head may be considered the changes wrought by removal to another country or climate.

The American dentist frequently has as patients foreigners that claim that, before immigrating, their teeth were in excellent condition. I call to mind among others a Swedish girl. She looked the picture of health. Her teeth were well-arranged and had ample room. Yet extensive decay was manifesting itself, though she had been only two years in America. Prior to her coming here she was blissfully unconscious of having a chewing-apparatus. Another patient, American, while in Paris pursuing art studies, had to make frequent visits to the dentist. When she went abroad, her teeth, I know, were in good repair. The French dentist informed her that she was sharing in the general experience of Americans sojourning in Paris.

These are of course individual cases, and should not be unreservedly taken as demonstrating a general fact. A grain of allowance must here be made for the complete change in the conditions of life. Then, too, worry and anxiety probably enter into consideration.

So far as I know, there is nothing in any country or climate that will act in a directly harmful manner upon the teeth. It is the *change* incident to removal that may, through the general system, result in increased liability to dental affections.

It is well known that the children of European parentage in India have a very low degree of physical vigor. No doubt had these children been born in Europe they would have probably been equally vigorous as their parents. But the climate itself of India must not be thought a cause of physical weakness. Fine development and good teeth are found in the aboriginal population of any inhabitable country. It is the persons "not to the manner born," or such as hereditarily belong to another country, that are likely to fail in health, and through this, as has been observed, in their teeth.

*Prenatal Influences.*—Much importance is attached, and justifiably so, to the attainment of vigorous health for women that are approaching motherhood. The heritage will be strong teeth and a strong systemic support. Dr. Rich, who has devoted much attention to the physical culture of women, observes, "Experiments have been made under the auspices of the French government, which proved conclusively that by increasing the health of the mother during pregnancy a marked effect is produced upon the teeth of the child. Fine teeth depend upon fine health of the mother. The benefits of vigorous exercise, proper diet, and the means of promoting a high condition of health are well known. Look at the mothers in our highly-civilized community, and what are they? They are delicate in frame, poorly developed, with soft muscles. Who ever heard of a fine horse being produced by a miserable dam? There may be a scalawag father, but it is always necessary to have a fine dam in order to produce a fine horse. This principle exists throughout the whole animal kingdom, not excepting man." Dr. Dwinelle, another authority in matters dental, believes that the child comes into the world with too little phosphate of lime in his system, resulting eventually in poorly calcified teeth. He thinks that to obviate this defect the mother should receive a saturating phosphate treatment. For substantiation he cites illustrative cases in his practice.

Dr. Rich's opinion, if the necessary provision for hereditary influences (paternal as well as maternal) be made, is no doubt a sound one, and in thorough accord with natural conservation. The implied advice is conservative, in that natural—I may say physiological—methods are resorted to. The doctor places, however, too much of the blame for poor teeth on the mother. The father, too, is partly responsible for defective dental heritage.

*Crossing.*—Dr. Brinton, an eminent ethnologist, in speaking of mulattoes, says, "It is well ascertained in the United States that they are peculiarly prone to scrofula and consumption, unable to bear hard work, and shorter lived than either the full black or the white. This is not owing to our climate, as the same results are recognized by the negroes of the Gold Coast, who for four hundred years have been in constant contact with the whites. In the West India Islands the mulattoes must be constantly reinforced by new crossings, or they disappear."

Such is the effect upon the general system. In a limited way the crossing of races, and of nationalities, will result in defective or irregular teeth. This observation has been made in the offspring of white and Indian parentage. The same may be said of the cross between the black and the white races. Even the teeth of children resulting from a union of Romanic and Germanic blood will often show, if not deteriorated tissual formation, at least irregularity of position, and that, as is well known, may lead up to positive lesion.

If we except the probable results of racial differences in the size of the jaws and the teeth of the parents, it is not likely that a cross between two pure types of races of sound physical development, living and brought up in practically the same climate, will have defective dental organs. In these days, however, such a case as just supposed would be hypothetical; most assuredly so as applied to white races.

The latter crossed with other races will undoubtedly confer upon the offspring a better brain-capacity than the inferior type possessed, but at the expense of a lowered physical development. It seems to show that civilized white man has removed himself quite far from nature. Since, as soon as he comes in contact with peoples nearer his condition, he entails upon them the physical penalties of an artificial life.

This ends the consideration of the remote factors.

But little need be said of the *immediate factors* in dental deterioration. They are *want of cleanliness*, *abuse*,—as in cracking hard nuts, and in similar feats of sleight of teeth; much *lemon-sucking*, and *eating* of *vinegar pickles* (school children offend in this way); *excessive indulgence* in acid phosphate; *acid medicinal preparations*, as lime-juice used as a gargle or the careless administration of *the chloride of iron tincture.*

The *eating* of *sweets* in moderation is harmless, but carried so far that the stomach is soured thereby, or the appetite for necessary food is taken away, it may result in indirect, if not direct, mischief to the dental organs. *Sudden thermal changes*, such as the teeth are subjected to at the table, may become injurious to the enamel, if not to the pulp as well. It must be hardy material, indeed, that can stand the taking of a gulp of ice-water on a gulp of hot soup. *Mouth-washes* and *tooth-powders* that are recommended to radically whiten the teeth and prevent decay are a source of harm. Professor Elliott is said to have analyzed one of these washes, and found it to contain eighteen per cent. concentrated *hydrochloric acid.* Another beautifying preparation is: water, one ounce; honey, two ounces; and *muriatic acid*, *one ounce*,—that is, twenty-five per cent. hydrochloric acid. The naïve directions are: "Mix, apply with a tooth-brush, and *rub vigorously*." If this does not whiten teeth, nothing short of extraction or death of the patient will.

To briefly summarize: Man owes the deterioration of his teeth (and, in a measure, of the related parts) to change in the mode of life; to change in diet, both as to character and preparation; to reduced general physical vigor; to weakening hereditary or peculiar maternal influences; to luxury, indolence, vice, capricious appetites, sleep-robbing, brain-fretting; to local abuse,—in general, to the sins of omission and commission against hygiene; against the conditions that necessitate the presence of teeth.

Dr. Hammond, in a fit of pessimism, predicts that, at the present rate of increase in baldness, a thousand years hence the human race will be hairless. He might have added that, if the miller continues to improve the fineness of flour; if the cook continues to improve in the art of making dishes for the palate only, and to multiply the number of highly-seasoned, stomach-teasing compounds called "fancy dishes;" if the kitchen-stove continues to do our chewing for us; if we continue to despatch our food by the watch, and, instead of insalivating the morsel, send it down under convoy of iced water or scalding coffee; if we continue to limit our physical exercise to the rocking-chair or to the office-stool; if we continue to shun the sunshine and crawl out after night-fall; if we continue to let, as Shakespeare says, "sweaty haste . . . make the night joint-laborer with the day," a thousand years hence our successors will be (I won't say toothless, that's nonsense, but) practically without bicuspids and molars, and the wearing of plates or bridges will have become as common as the wearing of shoes.

One of the Alfonsos of Spain is quoted as saying that if he had been consulted in the making of the world, better work would have resulted. I suppose if he had been called upon to assist in a palingenesis of the world, he would have either made man with an indurated gum to do duty for teeth, or constructed the latter so that when one is lost another would take its place in some such way as lizard-tails and crab-claws are reproduced. However, as Alfonso was *not* consulted, we shall be obliged to make the best of our lot, and—*let the dentist live.*

